# A Multi-Institutional Experience in Pediatric High-Grade Glioma

**DOI:** 10.3389/fonc.2015.00028

**Published:** 2015-02-18

**Authors:** Steve Walston, Daniel A. Hamstra, Kevin Oh, Gary Woods, Michael Guiou, Randal S. Olshefski, Arnab Chakravarti, Terence M. Williams

**Affiliations:** ^1^Department of Radiation Oncology, The Ohio State University Wexner Medical Center, Columbus, OH, USA; ^2^Department of Radiation Oncology, University of Michigan Health System, Ann Arbor, MI, USA; ^3^Department of Radiation Oncology, Massachusetts General Hospital, Boston, MA, USA; ^4^Department of Hematology-Oncology, Nationwide Children’s Hospital, Columbus, OH, USA

**Keywords:** high-grade glioma, glioblastoma, pediatric cancer, chemotherapy, radiation therapy

## Abstract

**Introduction:** Pediatric high-grade gliomas are rare tumors with poor outcomes and incompletely defined management. We conducted a multi-institutional retrospective study to evaluate association of clinical, pathologic, and treatment characteristics with outcomes.

**Materials and methods:** Fifty-one patients treated from 1984 to 2008 at the Ohio State University or University of Michigan were included. Histologic subgroups were compared. Log-rank and stepwise Cox proportional hazard modeling were used to analyze progression-free survival (PFS) and overall survival (OS) within the whole group, grade III subgroup, grade IV subgroup, and sub-total resection/biopsy subgroup.

**Results:** Median OS was 27.6 months. Grade III histology, complete tumor resection, and cerebral tumor location correlated with improved PFS and OS. Temozolomide use and chemotherapy after radiotherapy or chemoradiation (CRT) were associated with better PFS while seizure at presentation was associated with better OS. In multivariate analysis, complete resection and chemotherapy following radiotherapy or CRT were independent predictors for improved PFS and OS. For grade III and IV subgroups, complete resection was associated with improved OS (grade III) and seizure presentation was associated with improved OS (grade IV). In the incompletely resection subgroup, temozolomide use and concurrent CRT independently correlated with improved PFS, while higher radiation dose (≥59.4 Gy) and adjuvant chemotherapy were independently associated with improved OS.

**Discussion:** Total resection and receiving chemotherapy adjuvant to radiation or CRT are most closely associated with improved PFS and OS. For higher risk incompletely resected patients, temozolomide use and treatment intensification with concurrent CRT, adjuvant chemotherapy, and higher radiation dose were associated with improved outcomes.

## Introduction

High-grade gliomas (HGG) consist of grade III or IV tumors arising from glial cell origin, including anaplastic astrocytoma, anaplastic oligodendroglioma, glioblastoma, mixed glioma (1). In children, the incidence is approximately 0.85 per 100,000 population and they are generally estimated to account for 8–12% of childhood brain tumors (1, 2). When brainstem tumors are excluded, the location is most commonly supratentorial with about 35–50% of these tumors arising within the cerebral hemispheres (2, 3). Less commonly, tumors occur in the thalamus, hypothalamus, third ventricle, and basal ganglia (2, 3). The standard of care remains multimodal, utilizing a combination of surgery, radiation therapy (RT), and chemotherapy (2, 4). However, despite aggressive treatment, survival remains poor with a 5-year overall survival (OS) for anaplastic astrocytoma and glioblastoma typically ~30 and 15–20%, respectively (1).

The current treatment paradigm includes maximal safe resection as the primary treatment (2). Several prospective and retrospective series have highlighted the prognostic importance of maximizing extent of surgical resection (2, 5–7). Furthermore, midline tumor location rather than posterior fossa or cerebral hemisphere location has been associated with a worse prognosis perhaps due to decreased accessibility and extent of resection (6). Due to the infiltrative nature of HGG, a true complete resection, even when radiographic resection is evident, is unlikely and local recurrence is the dominant pattern of failure (2, 3, 8). Therefore, adjuvant treatment including RT and chemotherapy remain important for improving outcomes as further widening of surgical margins would result in unacceptable morbidity (2, 3, 6, 8). In addition, the value of temozolomide with RT has not been clearly established with randomized data in pediatric HGG, despite frequent use based on extrapolation from benefit seen with adult glioblastoma (9). Other issues surrounding the proper management of pediatric HGG include the use of adjuvant chemotherapy after RT or chemoradiation (CRT), and the appropriate radiation dose.

Due to the rarity of pediatric HGGs, it is difficult to conduct single institutional analyses to assess whether certain patient, tumor, or treatment characteristics affect outcome. Thus, in order to validate existing prognostic factors and identify novel prognostic factors for pediatric HGGs, two institutional experiences were pooled and analyzed. To our knowledge, this represents the largest retrospective study of pediatric HGG.

## Materials and Methods

We conducted a multi-institutional, retrospective IRB-approved study of 51 pediatric patients with HGG who were consecutively treated at the Ohio State University or the University of Michigan between 1984 and 2008. Brainstem gliomas were omitted from the study due to lack of histological diagnosis. Pre-treatment (patient), tumor, and treatment characteristics were collected on all patients from review of electronic and paper charts. Patient, tumor, and treatment characteristics were evaluated for the whole cohort and were also broken down by histologic grade subgroups (Table 1). Categorical variables between grade III and IV subgroups were compared using the Fisher’s exact *t*-test. A univariate analysis (UVA) was performed on these characteristics to analyze for factors determining improved progression-free survival (PFS) and OS in the whole cohort and each histological subgroup (Table 2), as well as a subgroup consisting of non-gross total resection (GTR) patients (Table 3). Factors evaluated include age, gender, presenting symptoms, duration of symptoms, tumor location, tumor size, histologic tumor grade, year treated, extent of resection, radiation dose, and type and timing of chemotherapy. OS and PFS were determined using the Kaplan-Meier method. The log-rank test was used to identify factors associated with improved PFS or OS and *p* < 0.05 was considered significant. Patients lost to follow-up were censored. Multivariate analysis (MVA) was performed with Cox proportional regression using prognostic factors identified in the UVA (Tables 4 and 5). SPSS, version 20 (SPSS Inc., Chicago, IL, USA) was used to perform the statistical analyses.

**Table 1:** **Patient, tumor, and treatment characteristics for the entire cohort, grade III subgroup, and grade IV subgroup**.

Factors	Whole cohort (*n* = 51)	Grade III (*n* = 23)	Grade IV (*n* = 28)	Fisher exact *t*-test
Median age	13 (4−20)	13 (4− 18)	13 (5− 20)	NA
Median follow-up	19 (2−269)	31 (5− 269)	15.5 (2− 142)	NA
**Gender**
Male	55% (*n* = 28)	48% (*n* = 11)	61% (*n* = 17)	*p* = 0.41
Female	45% (*n* = 23)	52% (*n* = 12)	39% (*n* = 11)	*p* = 0.41
**Duration of symptom presentation**
≥6 weeks	35% (*n* = 18)	52% (*n* = 12)	21% (*n* = 6)	*p* = 0.73
<6 weeks	31% (*n* = 16)	39% (*n* = 9)	30% (*n* = 7)	*p* = 0.73
**Symptoms at presentation**
Headache	61% (*n* = 31)	61% (*n* = 14)	61% (*n* = 17)	*p* = 1.0
Seizure	31% (*n* = 16)	35% (*n* = 8)	29% (*n* = 8)	*p* = 0.76
Cranial nerve deficit	14% (*n* = 7)	13% (*n* = 3)	14% (*n* = 4)	*p* = 1.0
**Location**
Cerebrum	69% (*n* = 35)	78% (*n* = 18)	61% (*n* = 17)	*p* = 0.23
Thalamus	16% (*n* = 8)	9% (*n* = 2)	21% (*n* = 6)	*p* = 0.27
Midbrain	4% (*n* = 2)	4% (*n* = 1)	3.5% (*n* = 1)	*p* = 1.0
Cerebellum	6% (*n* = 3)	0% (*n* = 0)	11% (*n* = 3)	*p* = 0.60
Other	6% (*n* = 3)	9% (*n* = 2)	3.5% (*n* = 1)	*p* = 0.58
**Size**
Median (cm)	5 (2.5−10)	5 (2.5− 7)	5 (3− 10)	NA
**Extent of resection**
Biopsy only	18% (*n* = 9)	13% (*n* = 3)	21% (*n* = 6)	*p* = 0.49
STR	59% (*n* = 30)	65% (*n* = 15)	54% (*n* = 15)	*p* = 0.57
GTR	23% (*n* = 12)	22% (*n* = 5)	25% (*n* = 7)	*p* = 1.0
**Radiation and chemotherapy treatment**
Median dose of RT	59.4 Gy	59.4 Gy	59.4 Gy	NA
Concurrent CRT	51% (*n* = 26)	17% (*n* = 4)	79% (*n* = 22)	***p* < 0.0001**
CT after RT/CRT	57% (*n* = 35)	61% (*n* = 14)	54% (*n* = 15)	*p* = 0.77
Concurrent and adjuvant CT	35% (*n* = 18)	13% (*n* = 3)	54% (*n* = 15)	***p* = 0.003**
Temozolomide use	39% (*n* = 20)	4% (*n* = 1)	68% (*n* = 19)	***p* < 0.0001**

*NA, not applicable; GTR, gross total resection; STR, sub-total resection; RT, radiation therapy; CT, chemotherapy; CRT, chemoradiation*.

*Bold indicates significant value (*p* ≤ 0.05)*.

**Table 2:** **Univariate analysis for the entire cohort, grade III subgroup, and grade IV subgroup for PFS and OS**.

Factors	PFS			OS		
	Whole cohort	Grade III	Grade IV	Whole cohort	Grade III	Grade IV
	*p*-value	*p*-value	*p*-value	*p*-value	*p*-value	*p*-value
**Patient characteristics**
Age (≤13 vs. >13 years)	0.854	0.537	0.881	0.139	0.095	0.681
Gender (male vs. female)	0.843	0.601	0.471	0.632	0.805	0.282
Symptom duration (<6 vs. ≥6 weeks)	0.594	0.361	0.528	0.379	0.696	0.364
Headache (yes vs. no)	0.351	0.850	0.193	0.272	0.237	0.540
Seizure (yes vs. no)	0.097	0.463	**0.008**	**0.019**	0.340	**0.016**
Nerve deficit (yes vs. no)	0.131	0.718	0.082	0.139	0.541	**0.035**
**Tumor characteristics**
Grade IV vs. grade III	**0.041**	NA	NA	**0.011**	NA	NA
Location (cerebrum vs. other)	**0.029**	0.242	**0.045**	**0.002**	**0.040**	**0.044**
Size (>5 cm vs. ≤5 cm)	0.494	0.474	0.190	0.878	0.792	0.925
**Treatment characteristics**
Year treated (before 1997 vs. 1997 or after)	0.822	0.126	0.529	0.972	0.131	0.527
Extent of resection (STR/biopsy vs.GTR)	**0.026**	0.347	**0.031**	**0.004**	**0.006**	0.130
Concurrent CRT (yes vs. no)	0.759	0.656	0.997	0.786	0.412	0.491
CT after RT/CRT (yes vs. no)	**0.003**	0.905	0.681	**0.001**	0.211	0.589
Temozolomide (yes vs. no)	**0.032**	0.137	**0.006**	0.309	0.214	0.336
Concurrent and adjuvant CT (yes vs. no)	0.566	0.475	0.403	0.879	0.438	0.947
Radiation dose (≥59.4 vs. <59.4 Gy)	0.696	0.592	0.592	0.392	0.065	0.739

*NA, not applicable; STR, sub-total resection; GTR, gross total resection; CT, chemotherapy; RT, radiation therapy; CRT, chemoradiation*.

*Bold indicates significant value (*p* ≤ 0.05)*.

**Table 3:** **Univariate analysis for the STR/biopsy only subgroup**.

Factors	PFS			OS		
	Whole cohort	Grade III	Grade IV	Whole cohort	Grade III	Grade IV
	*p*-value	*p*-value	*p*-value	*p*-value	*p*-value	*p*-value
Grade IV vs. grade III	**0.041**	NA	NA	**0.011**	NA	NA
Concurrent CRT (yes vs. no)	0.851	0.617	0.470	0.769	0.940	0.697
CT after RT/CRT (yes vs. no)	**0.006**	0.545	0.257	**0.001**	0.162	0.980
Temozolomide (yes vs. no)	0.080	0.066	0.828	0.354	0.350	0.541
Concurrent and adjuvant CT (yes vs. no)	0.205	0.375	0.795	0.286	0.473	0.507
RT dose (≥59.4 vs. <59.4 Gy)	0.598	0.967	**0.018**	0.203	0.421	**0.027**

*NA, not applicable; CT, chemotherapy; RT, radiation therapy; CRT, chemoradiation*.

*Bold indicates significant value (*p* ≤ 0.05)*.

**Table 4:** **Multivariate analysis for the entire cohort and grade III/IV subgroups**.

	Adjusted HR	95% CI	*p*-value
**WHOLE COHORT**			
**Progression-free survival (PFS)**
Grade IV vs. grade III	0.573	0.278–1.181	0.131
Cerebrum vs. other	1.623	0.719–3.665	0.244
STR/biopsy vs. GTR	0.262	0.089–0.775	**0.015**
CT after RT/CRT (yes vs. no)	3.391	1.526–7.218	**0.002**
Temozolomide (yes vs. no)	1.513	0.746–3.068	0.251
**Overall survival (OS)**
Grade IV vs. grade III	0.540	0.241–1.210	0.135
Cerebrum vs. other	1.621	0.710–3.701	0.252
STR/biopsy vs. GTR	0.202	0.060–0.683	**0.010**
Seizure vs. no seizure	1.431	0.528–3.880	0.482
CT after RT/CRT (yes vs. no)	4.187	1.856–9.442	**0.001**

**Grade III**			
**Progression-free survival (PFS)**
Cerebrum vs. other	1.97	0.586–6.645	0.273
STR/biopsy vs. GTR	0.57	0.128–2.571	0.467
**Overall survival (OS)**
Cerebrum vs. other	1.81	0.519–6.286	0.353
STR/biopsy vs. GTR	0.16	0.034–0.743	**0.019**

**Grade IV**			
**Progression-free survival (PFS)**
Seizure vs. no seizure	3.26	0.969–10.991	0.056
Cranial nerve deficit (yes vs. no)	0.38	0.110–1.316	0.127
Cerebrum vs. other	1.52	0.625–3.716	0.354
STR/biopsy vs. GTR	0.64	0.143–2.887	0.564
Temozolomide (yes vs. no)	2.49	0.896–6.898	0.080
**Overall survival (OS)**
Seizure vs. no seizure	4.20	1.181–14.969	**0.027**
Cranial nerve deficit (yes vs. no)	0.57	0.133–2.009	0.383
Cerebrum vs. other	1.75	0.613–4.969	0.297
STR/biopsy vs. GTR	0.99	0.216–4.520	0.988
Temozolomide (yes vs. no)	1.24	0.418–3.705	0.694

*STR, sub-total resection; GTR, gross total resection; CT, chemotherapy; RT, radiation therapy; RT, chemoradiation*.

*Bold indicates significant value (*p* ≤ 0.05)*.

**Table 5:** **Multivariate analysis for the STR/biopsy only subgroup**.

	Adjusted HR	95% CI	*p*-value
**Progression-free survival (PFS)**
Grade IV vs. grade III	0.48	0.220–1.034	0.061
RT dose (≥59.4 vs. <59.4 Gy)	1.28	0.445–3.687	0.647
Temozolomide (yes vs. no)	8.81	2.397–32.401	**0.001**
CT after RT/CRT (yes vs. no)	1.61	0.581–4.443	0.361
Concurrent CRT (yes vs. no)	6.25	1.75–23.809	**0.005**
Concurrent and adjuvant CT (yes vs. no)	2.83	0.812–9.892	0.102
**Overall survival (OS)**
Grade IV vs. grade III	0.42	0.173–1.004	0.051
RT dose (≥59.4 vs. <59.4 Gy)	2.84	1.129–7.137	**0.027**
Temozolomide (yes vs. no)	1.72	0.761–3.862	0.193
CT after RT/CRT (yes vs. no)	2.67	1.093–6.541	**0.031**
Concurrent CRT (yes vs. no)	1.72	0.477–6.173	0.408
Concurrent and adjuvant CT (yes vs. no)	1.43	0.301–6.822	0.651

*CT, chemotherapy; RT, radiation therapy; CRT, chemoradiation*.

*Bold indicates significant value (*p* ≤ 0.05)*.

## Results

### Patient characteristics and survival

**Figure 1:**
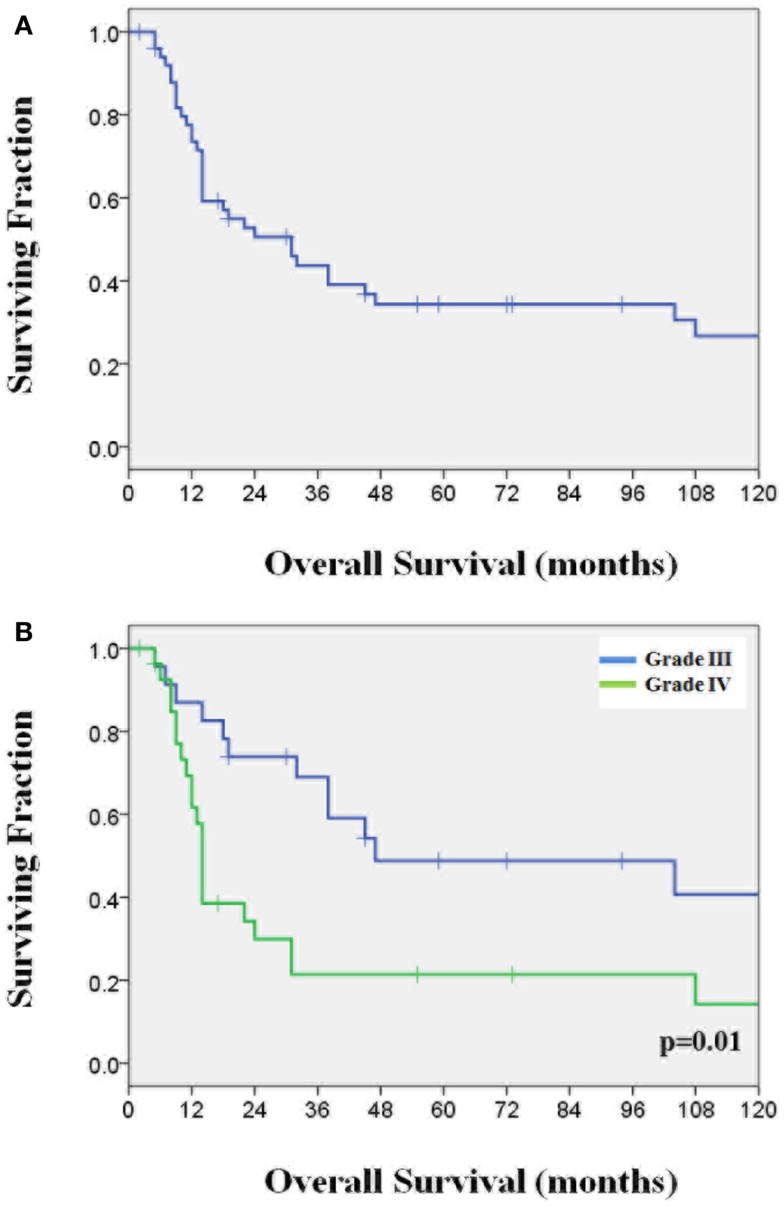
**(A)** Overall survival for the whole cohort; **(B)** Overall survival for the grade III and grade IV subgroups.

Median follow-up for the whole cohort was 19 months from diagnosis. The 1- and 2-year OS rates were 78 and 53%, respectively, and the median survival (MS) was 27.6 months (Figure 1A). Table 1 shows patient characteristics for the whole cohort, as well as for grade III and grade IV tumors, with statistical comparisons between grade III and IV tumor groups. Histologic distribution was 45% (*n* = 23) grade III and 55% (*n* = 28) grade IV. All patients were under 21 years old with a median age at diagnosis of 13 years (range 4–20) and 55% were males. Tumor location was cerebrum (*n* = 35), thalamus (*n* = 8), midbrain (*n* = 2), cerebellum (*n* = 3), and other (*n* = 3; foramen of Monro/ventricle, suprasellar, and not available). All patients received at least CT scans for staging, 82% received MRI staging and post-treatment MRI scans. Analysis for duration of symptom presentation was dichotomized at the median of 6 weeks. The most common presenting symptoms were headache 61% (*n* = 31), seizure 31% (*n* = 16), and cranial nerve deficit 14% (*n* = 7). Other factors were also dichotomized by the median for the UVA including age (13 years), tumor size (5 cm), year treated (1997), and radiation dose (59.4 Gy).

Extent of surgical resection was defined by the surgeon, post-operative imaging, or both in all cases and consisted of GTR 23% (*n* = 12), sub-total resection (STR) 59% (*n* = 30), and biopsy only 18% (*n* = 9). All patients received RT within 4 weeks following resection and the median dose for the whole cohort was 59.4 Gy in 33 fractions (range, 41.4–72 Gy in 23–60 fractions). In the analysis, radiation dose was dichotomized by <59.4 Gy (*n* = 21 patients) vs. ≥59.4 Gy (*n* = 30 patients). An almost equal percentage of patients in the grade III and grade IV groups received at least 59.4 Gy [grade III 61% (*n* = 14), grade IV 57% (*n* = 16)]. Forty-two (82%) patients received chemotherapy as part of treatment either concurrent with radiation only 51% (*n* = 26), after RT or CRT 69% (*n* = 35), or both 35% (*n* = 18). Twenty patients (39%) received temozolomide as part of their chemotherapy [31% (*n* = 16) concurrent and adjuvant, 4% (*n* = 2) concurrent only, 4% (*n* = 2) adjuvant only], eight (16%) patients received concurrent vincristine, six patients (12%) received adjuvant vincristine, lomustine, and prednisone (pCV), and five (10%) patients received adjuvant procarbazine, lomustine, and vincristine (PCV). Alternate chemotherapy regimens were used in less than 10% of the patients. The median number of adjuvant chemotherapy cycles was seven.

In the grade III subgroup, the median age was 13 years, with a median follow-up of 31.0 months. The MS was 31.0 months, and the 1- and 2-year survival rates were 87 and 59%, respectively (Figure 1B). In the grade IV subgroup, median age was 13 years, with a median follow-up of 15.5 months. The MS for grade IV tumors was 22.9 months, with 1- and 2-year OS rates of 71 and 48%, respectively (Figure 1B). Furthermore, the OS difference between grade III and IV was statistically significant (*p* = 0.011) with UVA. As shown in Table 1, patients with grade III tumors received aggressive treatment regimens significantly less often with lower rates of concurrent CRT [17% (*n* = 4) vs. 79% (*n* = 22); (*p* < 0.0001)] and concurrent CRT plus adjuvant chemotherapy [13% (*n* = 3) vs. 54% (*n* = 15); (*p* = 0.003)]. However, those with grade III histology received adjuvant chemotherapy after RT or CRT with a similar frequency compared to grade IV patients [grade III 61% (*n* = 14); grade IV, 54% (*n* = 15)]. Temozolomide use was significantly less common in grade III patients compared to grade IV patients [4% (*n* = 1) vs. 68% (*n* = 19); (*p* < 0.0001)].

### Analysis of prognostic factors in whole cohort

The UVA for the whole group and each histologic subgroup is shown in Table 2. In the whole cohort, factors that significantly predicted for improved PFS were: grade III histology, cerebral location, GTR, receiving chemotherapy after RT/CRT, and receiving temozolomide as part of their chemotherapy regimen. The factors that predicted for significantly improved OS were identical to PFS, except use of temozolomide was not significant. Additionally, seizure at presentation predicted for improved OS, where there was only a trend in the PFS analysis. A MVA was performed using prognostic factors identified in the UVA (Table 4). The only factors that maintained significance for PFS were GTR [*p* = 0.015, HR 0.26 (95% CI 0.09–0.78)] and not having received chemotherapy after RT/CRT [*p* = 0.002, HR 3.39 (95% CI 1.53–7.22)]. The same factors were independent predictors for improved OS: GTR [*p* = 0.01, HR 0.20 (95% CI 0.06–0.68)] and not having received chemotherapy after RT/CRT [*p* = 0.001, HR 4.19 (95% CI 1.86–9.44)]. Interestingly, histologic grade was not found to be an independent predictor of PFS or OS.

### Subgroup analysis of prognostic factors by grade or extent of resection

Within the grade III subgroup, UVA revealed no factors with significant impact on PFS, but cerebral location and GTR significantly predicted for improved OS (Table 2). MVA demonstrated that GTR was the only factor which continued to show significance for improved OS [*p* = 0.019, HR 0.16 (95% CI 0.034–0.74), Table 4]. Within the grade IV subgroup, several factors were significant for improvement in PFS and OS with UVA. Seizure at presentation, cerebral location, GTR, and treatment with temozolomide were associated with improved PFS, while seizure at presentation, absence of cranial nerve deficit at presentation, and cerebral location were associated with improved OS. MVA found no associations with these factors for improved PFS, and only lack of seizure at presentation was significantly associated with worse OS compared to those who presented with seizure [*p* = 0.027, HR 4.20 (95% CI 1.18–14.97), Table 4].

The majority of the patients in this study, 77% (*n* = 39), did not receive a GTR and an additional subgroup analysis was performed on these patients. Of these, 23% (*n* = 9) underwent biopsy only and 77% (*n* = 30) received STR. Given the strong prognostic value associated with GTR, we hypothesized that certain treatment variables such as chemotherapy or radiation could impact PFS or OS in the setting of incomplete resection. In this group, the median RT dose was 59.4 Gy, 51% (*n* = 20) received concurrent CRT, 59% (*n* = 23) received chemotherapy after RT/CRT, 41% (*n* = 16) received both concurrent and adjuvant chemotherapy, and 38% (*n* = 15) received temozolomide. UVA was performed on grade and treatment factors in this STR/biopsy subgroup (Table 3). As previously noted with the whole cohort analysis, grade III patients and patients who received chemotherapy following RT/CRT had significantly improved PFS and OS. When focusing on the grade III patients within the STR/biopsy subgroup, no factors were significant for PFS or OS. For the grade IV patients within the STR/biopsy subgroup, the only significant factor was radiation dose, with those patients receiving either the median dose (59.4 Gy) or higher demonstrating improved PFS and OS. Finally, a MVA was performed on the STR/biopsy subgroup with these same treatment factors and tumor grade (Table 5). Treatment with temozolomide or concurrent CRT was significantly associated with improved PFS, while higher radiation dose and chemotherapy given after RT/CRT were significant for improved OS.

## Discussion

Although MS is typically higher than that of adults, outcomes for pediatric HGGs remain poor. Due to their rarity, studies directed at identifying prognostic, clinical, or pathological factors and optimal treatment strategies are difficult. Consequently, adult treatment strategies, such as the use of concurrent temozolomide and RT, are often extrapolated to children even though there is data indicating these diseases are biologically distinct (10). By pooling institutional datasets, our study represents the largest retrospective evaluation of pediatric HGGs to our knowledge, which has facilitated improved detection of prognostic factors and also hypothesis-generating subgroup analyses.

Comparing our institutional results to others, the OS in our patients appears to be similar to recent randomized studies in pediatric HGG. As with our study, these trials typically group both grade III and grade IV patients together. For example, the MS and 5-year OS for CCG-945 and our study were 26 vs. 27.6 months and 36% vs. 34%, respectively (6). Thus, our dataset is applicable to pediatric patients treated in the current era. Our analysis revealed that certain treatment variables, including extent of resection, concurrent and adjuvant chemotherapy, and radiation dose were prognostic of outcomes in the whole group or in subgroup analyses. We elaborate on these factors below.

Although randomized controlled trials for degree of resection are unlikely to be conducted, a more complete resection has been correlated with improved PFS and OS in numerous studies (2, 5–7, 11). This finding was corroborated in our study. Achieving a GTR is associated with a significant improvement in both PFS and OS in the total group, improved PFS in the grade IV subgroup, and OS in the grade III subgroup. Furthermore, tumors involving the cerebral hemispheres were associated with an improved OS in all three populations (whole cohort, grade III, and grade IV), which could relate to better accessibility for complete resection (6). Cerebral hemisphere location also significantly improved PFS in the grade IV patients as well as the whole cohort. However, on MVA, GTR retained significance for PFS and OS, while tumor location did not. This result was recently observed in a retrospective study which consisted of 27 pediatric grade IV patients: both tumor location and extent of resection were significant on UVA, but only extent of resection was independently correlated with OS on MVA (11).

The use of any adjuvant chemotherapy, following RT/CRT improved PFS and OS in both univariate and multivariate analyses for the whole group. However, chemotherapy drug regimens varied widely in our population. Despite this, 82% of patients (*n* = 42 total; 65% of grade III patients, 96% of grade IV patients) did receive chemotherapy, with 39% (*n* = 20) receiving temozolomide. It is generally assumed that the addition of chemotherapy after radiation improves outcome, but this has not been established in a prospective, randomized trial. In CCG-943, CRT with vincristine followed by 48 weeks of pCV vs. radiation alone resulted in improved 5-year event-free survival of 46% vs. 18% (5). However, this randomized trial altered two variables by adding both concurrent and adjuvant chemotherapy, which prevented the determination of independent benefits for either adjuvant or concurrent chemotherapy. In our cohort, temozolomide was a frequent component of treatment for grade IV patients with 68% (*n* = 19) receiving this drug either concurrent with radiation only (*n* = 2), adjuvant to radiation only (*n* = 2), or both (*n* = 15). Comparatively, only 4% (*n* = 1) of grade III patients were treated with temozolomide. Interestingly, there was significantly improved PFS for the patients who received temozolomide in both the total group and grade IV subgroup, though this was not significant on MVA. No OS advantage was observed, which was possibly due to the significant grade imbalance: 95% of the patients who received temozolomide had grade IV histology which predicted for worse OS on UVA. In ACNS0126, a single-arm phase II study, using concurrent and adjuvant temozolomide in pediatric patients with grade IV glioma, no benefit was detected in PFS or OS when compared to the CCG-945 patients who received either pCV-based CRT or eight-drugs-in-one-based therapy (12). However, due to reduced toxicity compared to other regimens, temozolomide-based treatment has been widely adopted and will likely be used as the platform in future trials. It is important to recognize MGMT overexpression is a negative prognostic factor for HGG and temozolomide may be more effective in those patients with decreased enzyme expression or promoter methylation (13, 14). One potential limitation of our study was that MGMT status was not available in our analysis. Taken together, our data suggest that adjuvant chemotherapy and/or temozolomide may improve outcomes.

In our study, 51% of patients received concurrent CRT compared to radiation alone. The benefit of adding chemotherapy to radiation in a concurrent fashion compared to radiation alone has also not been directly tested in pediatric trials. Since concurrent CRT is the present standard of care, it is unlikely a trial enrolling an arm seemingly inferior to the standard will ever be tested (2). However, despite the improved outcome with combined modality treatment demonstrated in previous randomized trials, concurrent CRT did not show a benefit in our retrospective cohort (4, 5). In addition, there was no difference noted in outcomes for the 35% of patients who received both concurrent and adjuvant chemotherapy (which is currently the standard of care for adult and pediatric HGG patients) (2, 9). A possible explanation for this lack of association is that the CRT group was enriched with negative prognostic variables including STR/biopsy (77%) and grade IV histology (85%), and the patients given CRT followed by adjuvant chemotherapy were composed of 86% grade IV tumors. Even with these imbalances, no differences were observed in PFS or OS. Thus, it would appear in this limited analysis, that these intensified treatment regimens might have acted to offset the imbalances between the grade III and grade IV groups, resulting in equivalency in outcomes.

Adjuvant radiation remains a standard of care due to the invasive nature of HGGs, high local failure rate after surgery, and morbidity with more extensive surgery in the brain (4). Typically, 50–60 Gy has been delivered to pediatric patients after surgery, with the majority of clinical trials using a dose of 54 Gy in 30 fractions (5, 6, 12). Efforts to improve outcomes with hypo- or hyper-fractionation have been unsuccessful or have not consistently shown benefit (2, 15). Every patient in our study received adjuvant RT with a median dose for the whole cohort and all subgroups of 59.4 Gy. We found receiving a dose at or above the median dose of 59.4 Gy was not associated with benefit in grade III, grade IV, or the whole cohort. Interestingly, for the subgroup of patients with incomplete resection, radiation dose at or above the median dose of 59.4 Gy did show a significant improvement in PFS and OS in the grade IV patients. Moreover, the higher dose of radiation remained significant for improved OS with MVA. As a result of STR, these patients are at higher risk for local recurrence. Therefore, it is attractive to speculate that escalation of local therapy could offer improvement in outcomes. As suggested by our findings, it may be prudent to escalate the dose to at least 59.4 Gy for grade IV patients who are unable to achieve a GTR.

In general, grade IV tumors tend to have a worse PFS and OS when compared with grade III tumors, and the majority of pediatric trials have included both histologies in their treatment schema (2, 5, 6, 12). As compared with grade III disease, grade IV tumors are more rapidly proliferating, demonstrate a more aggressive infiltrative pattern, and may be more treatment resistant, which all portend a worse prognosis. Our results are commensurate with previous reports, as patients with grade IV histology had decreased PFS and OS compared with grade III, though this did not reach significance on MVA. However, in patients who had an incomplete resection, lower tumor grade was associated with improved PFS on UVA and significantly improved OS on UVA and MVA. Patients presenting with a seizure had a better OS on UVA in the whole cohort and the grade IV subgroup and this remained significant for the grade IV subgroup. It is possible these patients had less aggressive biology or more favorable tumor location as 57% of grade IV patients presenting with seizure had GTR while only 25% of all grade IV patients had a GTR.

In summary, GTR and administration of chemotherapy after RT/CRT were found to be the most significant predictors for PFS and OS, each remaining significant on MVA for the whole group. Interestingly, grade was not significant for the whole group on MVA, perhaps since grade IV patients were enriched with patients who received temozolomide and treatment intensification, including concurrent CRT or concurrent and adjuvant chemotherapy. Thus, the more intense treatments may have served to equalize outcomes between grade III and grade IV tumors. In non-GTR patients, adjuvant chemotherapy appeared to significantly improve outcomes in MVA. Finally, in the grade IV patients within the STR/biopsy subgroup, dose escalation to ≥59.4 Gy significantly improved OS on MVA. Due to its retrospective design, there are inherent limitations to this study which make it possible that certain outcomes are subject to confounding factors that were unaccounted for. Specifically, we were unable to control for selection bias, accurate reporting of toxicities, low patient numbers, performance status, MGMT methylation status, or other genetic factors that would be better accounted for in prospective trials. Also, there was no central pathologic review, which has been shown in several CCG/COG studies to significantly alter histological designation (16). Furthermore, even though treatment in different eras did not appear to impact outcome using a dichotomous variable (Table 2), it remains possible that there was variation in diagnostic evaluation, histologic definition/criteria, radiation/surgical technique, chemotherapy, or quality of supportive care which may have impacted outcomes. Thus, additional validation of these prognostic factors prospectively and/or in larger datasets is warranted. Future clinical studies building upon a regimen that includes radiation with concurrent and adjuvant TMZ could consider stratifying by molecular factors, extent of resection, grade, and potentially the other risk factors elucidated here. Our results suggest that efforts to enhance tumor resection in order to achieve GTR, possibly through improved pre-surgical imaging techniques or intraoperative brain mapping, may improve outcome. Furthermore, adjusting the length and/or type of adjuvant chemotherapy or treatment intensification in high-risk groups (possibly with radiation dose escalation) could be valid directions for further study.

## Conflict of Interest Statement

The authors declare that the research was conducted in the absence of any commercial or financial relationships that could be construed as a potential conflict of interest. The Associate Editor Minesh P. Mehta declares that, despite having collaborated with Arnab Chakravarti, the review process was handled objectively and no conflict of interest exists.
